# Therapeutic use of cannabis in the US

**DOI:** 10.1097/01.NPR.0000884880.81603.c5

**Published:** 2022-11-22

**Authors:** Tracy A. Klein, Carey S. Clark

**Affiliations:** **Tracy A. Klein** is Assistant Director at the Center for Cannabis Policy, Research, and Outreach and an associate professor at College of Nursing, Washington State University Vancouver in Vancouver, Wash.; **Carey S. Clark** is Professor of Nursing and Medical Cannabis at Pacific College of Health and Science, San Diego, Calif.

**Keywords:** cannabis, marijuana, medical marijuana, NP

## Abstract

NPs are likely to encounter patients using cannabis with therapeutic intent, with or without legal authorization. During the clinical history and assessment process, NPs need to engage in frank discussion about cannabis therapeutics, including the risks and benefits, evidence for use, dosing considerations, potential drug interactions, and harm reduction.

**Figure:**
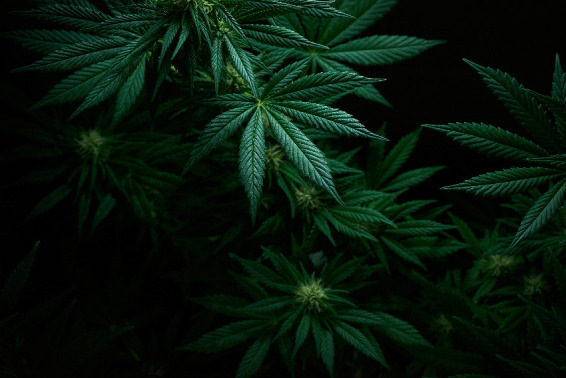
No caption available.

In 2021, 17 states and the District of Columbia specified that NPs could authorize patients for medical cannabis use, and as of January 1, 2022, NPs in Oregon can also authorize patients for medical cannabis.^1^-^3^ In 2022, a total of 37 states, 4 territories, and the District of Columbia allow medical cannabis use for authorized conditions.^4^ The prevalence of public and legislative support for use of cannabis for medical conditions as well as the state tax revenue produced by its sale creates a favorable environment for continued expansion of medical cannabis programs.^5^ NPs are therefore likely to encounter, counsel, and treat patients who are using cannabis medically (for therapeutic use), with or without healthcare provider authorization, as well as patients who use cannabis “recreationally” who may also be interested in taking a more therapeutic approach to their cannabis use.

In multiple studies, healthcare professionals have self-assessed that their knowledge of cannabis pharmacology, dosing, and clinical application is poor.^6^-^8^ NPs are open to education about cannabis and continuing education regarding cannabis. Even if NPs do not intend to authorize or recommend cannabis for medical use, evidence-based information can make NPs more likely to inquire about their patients' cannabis use and provide education and coaching regarding critical pharmacologic impact such as drug-drug interactions, proper dosing, and potential adverse reactions.^9^ The following article offers background on therapeutic cannabis and its use in the US, which may facilitate NP discussion of cannabis with patients and help guide cannabis use assessment in patient encounters. Therapeutic use is primarily defined as use with healthcare practitioner authorization; however, the authors acknowledge that due to stigma, cost, and other barriers, patients may use cannabis with therapeutic intent without having state authorization. While used therapeutically by patients, it is important to note that at this time nonpharmacologic cannabis and cannabinoid products are not FDA approved. The American Nurses Association supports the protection from civil, criminal, and professional penalties for nurses who discuss, authorize, or counsel patients regarding cannabis as a medical alternative.^10^

## The endocannabinoid system

Cannabinoids can be plant-based, synthesized, or produced within the body. The endocannabinoid system (ECS) is an organized system throughout the body containing receptors that respond to endogenously produced cannabinoid ligands; phyto- and synthetic cannabinoids; and biosynthetic and degradative enzymes.^11^ The ECS is a neuromodulatory system that is also capable of biosynthesizing endocannabinoids on demand.^12^ While commonly defined as consisting of CB1 and CB2 receptors and their activity, the actions of the endocannabinoid system are more complex and interactive. Additionally, the majority of cannabinoids do not act on CB1 receptors which are primarily found in the central nervous system and bound with a high affinity by the cannabinoids tetrahydrocannabinol (THC) and delta-9-tetrahydrocannabivarin (THCV).^13^ It is also important to note that endocannabinoid-like mediators have targets that both overlap and differ structurally from the phytocannabinoids.^13^ This complexity supports the adage that the whole is more than the sum of its parts, leading to therapeutic research exploring how targeting cannabinoid receptors can selectively and efficiently treat illness without upsetting the inherent ECS homeostasis. The ECS influences responses throughout the body including anxiety, depression, neurogenesis, pain, hunger, and cognition.^14^ Cannabinoids are also thought to be potent anti-inflammatory agents which have the potential for use in managing a variety of conditions such as skin, gastrointestinal, and immune disorders.^14^

## Cannabis pharmacology

There are now over 700 cannabis chemovars (the chemical composition fingerprint of each cannabis plant) containing hundreds of active compounds including cannabinoids, which are the active constituents, and terpenes, which are the volatile compounds that create both flavor and odor.^1^,^15^ Cannabinoids exert their pharmacologic effect by binding to receptors found within the body's ECS. The two most studied major cannabinoids are delta-9-tetrahydrocannabinol (referred to as THC or delta-9-THC to differentiate it from other THC formulations) and cannabidiol (CBD).

When administered, cannabis is highly lipophilic. Its lipophilic nature has implications for both routes of administration as well as the length of effect in the body as compared with other drugs or substances such as alcohol. Effective administration for therapeutic effect involves either: a) distribution through inhalation which decarboxylates THC in the plant to a more potent and active form, or b) ingestion or administration in a lipid-soluble form or with a lipid-rich meal.^16^ Edibles also need to be decarboxylated to create THC's hedonic effects. The pharmacokinetics of other routes of administration are not well researched for therapeutic use; ingestion of capsules and tablets is the most commonly studied.^17^ THC is a partial agonist at the CB1 and CB2 receptors in the ECS where it has analgesic and psychoactive effects based on agonism of CB1 receptors.^18^ CBD has little binding affinity for CB1 and CB2 and is thought to have its effect primarily by binding with noncannabinoid receptors as well as having modulatory effects on CB1 receptors.^18^,^19^

## Pharmacokinetics

While there are over 100 other cannabinoids, this review will focus on THC and CBD and their use, with an understanding that patients using whole plant products may be exposed to other compounds in the cannabis product which also may have pharmacologic effects. Because cannabis can be used in a number of different preparations and ways, both the formulation and route of administration, as well as patient factors, impact its pharmacokinetics.^18^ The pharmacokinetic properties of cannabis are therefore dynamic and can change over time and be patient-specific.^16^

The metabolism of THC is predominantly hepatic, via the cytochrome P450 (CYP 450) isozymes CYP2C9, CYP2C19, and CYP3A4.^18^ CBD is also hepatically metabolized, primarily by isozymes CYP2C19 and CYP3A4 and additionally, CYP1A1, CYP1A2, CYP2C9, and CYP2D6.^20^

If inhaled by vaporization or by smoking, peak plasma concentrations of cannabinoids are achieved within 3-10 minutes with a bioavailability ranging from 10% to 35%. If smoked, about 40%-50% of THC is lost to noninhaled and exhaled smoke.^18^,^21^ While both THC and CBD are metabolized in the liver, inhalation avoids or significantly reduces the first-pass hepatic effects.^18^

Ingestion of cannabis in the edible form causes a peak plasma concentration of THC which may occur twice successively within 1—6 hours after ingestion.^22^ CBD and other plant cannabinoids have a similar bioavailability when ingested as THC, which is estimated to be about 6%-7%, and can be increased by taking it with a fatty meal.^22^ For medical use, legally sanctioned oils of various concentrations are available in Canada and other countries.^17^,^23^ Published studies on oral pharmacokinetics focus on synthetic forms and analogues of THC, without considering the other cannabinoids usually present in plant-derived products.^23^ Because CBD and other cannabinoids are present in many preparations, this may also alter the THC pharmacokinetics.^23^ Sublingual and other oral mucosal preparations have been developed for medical use and more rapid onset.^16^ CBD and THC both vary in their half-life when administered orally. The half-life of CBD is reported to be between 1.4 and 10.9 hours after oromucosal spray, 2-5 days after chronic oral administration, 24 hours after I.V., and 31 hours after smoking.^24^ The half-life for THC is variable and longer in individuals who use cannabis heavily regardless of how it is taken.^18^ Plasma half-life of THC is estimated to be 1-3 days in occasional users and 5-13 days in chronic users; however, orally administered pharmaceutical products have a much lower plasma concentration when tested as compared with smoked products.^16^,^25^

## Pharmacodynamics

The biochemical and physiologic effects of the drug have been studied on healthy volunteers and observed with individuals who use cannabis.^18^ Pharmacokinetic variability previously discussed such as length of time used, age, and body weight and composition may impact the pharmacodynamic effects of the drug on the user. Users of cannabis, as with most drugs, develop tolerance to effects over time due to pharmacodynamic adaptation.^16^ While the incidence of cannabis dependence is thought to be low compared with opioids, there is potential in vulnerable patients for cannabis use disorder. The true incidence of dependence potential is difficult to determine due to stigma and methodological issues.^26^ However, most estimates are that cannabis use disorder will affect about 9%-10% of cannabis users.^26^,^27^ The majority of impairing and pathologic effects are related to the THC content of cannabis, which can be balanced by titration, reduction in dosage, and addition of the balancing effects of CBD.^19^ While the toxicity of cannabis is low when used medically and judiciously, there is particular concern about toxicity potential in patients at risk by pattern of use, co-occurring disease or drug use, and/or age. Age and gender may also impact choice of cannabis products based on both user and marketing factors. Older adults who use cannabis do not necessarily limit their consumption to edible or topical formulations and often use a wide range of products.^28^ Lethargy and ataxia are the primary symptoms in children who have ingested cannabis, generally in resin, cookies/edibles, or cannabis cigarettes (joints).^29^ Use of high doses of THC-predominant cannabis (that is, over 10 mg per dose) or doses exceeding 20-30 mg daily by adults can increase adverse reactions and decrease efficacy and may suggest tolerance or misuse.^19^

## Drug-drug interactions

Drug interactions may occur based on the THC, CBD, or both in a cannabis product. CBD, which is also available over the counter in most states in the US, has known potential for many drug interactions which may go unrecognized due to its easier access and widely available status. However, it is also important to note that many drug interactions are theoretical and may not correlate with a clinical impact. Counseling of patients regarding drug-drug interactions should therefore be comprehensive and consider individual patient factors as well as the intended therapeutic use of cannabis. As an example, infrequent use for an intermittent or rare symptom may have less impact than frequent use for a chronic condition or as a substitute for a prescription medication. A drug interaction can also be additive, synergistic, or antagonistic and each drug interaction will have different effects.^30^ Opioids and/or their active metabolite levels are increased when taken along with cannabinoids, which enhances analgesic levels, and this is the usual desired effect.^31^ However, when cannabis is used with drugs such as morphine which can cause respiratory depression, a dose reduction may be required due to enhanced potency of both medications and the potential for negative effects.^31^ While current studies are primarily theoretical, observational, or case-based, there is a potential for augmented potency and therefore a recommendation for decreased dosing should be advised for morphine, codeine, oxycodone, methadone, and tramadol when used with cannabinoids.^31^ One double-blind study found that coadministration of a large dose of CBD (400-800 mg) with I.V. fentanyl did not potentiate adverse cardiovascular or respiratory effects and was well tolerated.^32^

Hepatic damage can result from the use of cannabinoids and valproic acid or acetaminophen due in part to THC and/or CBD inhibition of UDP glucuronosyltransferases—enzymes that help biotransform the drugs to less toxic components.^31^ Dose reductions may be required for antidepressants used for pain relief such as duloxetine, amitriptyline, and venlafaxine.^31^ Because they are renally excreted, there are no known drug interactions for pregabalin or gabapentin with cannabis.^31^ CBD has a clear interaction with clobazam, significantly increasing the levels of its active metabolite N-desmethylclobazam in several studies; this is felt to be due to CBD's inhibition of CYP2C19. Other data demonstrate possible interactions with rufinamide, zonisamide, topiramate, and eslicarbazepine.^33^ Additionally, potential drug interactions for CBD include many drugs which are used daily, including anticoagulants.^34^ While caution should be used in interpreting results, a publicly available drug interaction checker can be found at: www.drugs.com/drug-interactions/cannabis.html which lists minor to major potential drug interactions. Patients should be educated about potential drug interactions and symptoms to monitor.

## Indications

Indications for medical cannabis are legally authorized in the US by state law and vary from state to state. Pain, cachexia, cancer, posttraumatic stress disorder, seizure disorders, and nausea/vomiting are some of the most common conditions for which cannabis can be authorized medically in the US.^35^ A yearly summary of conditions by state is published and updated at: www.safeaccessnow.org/condition. Cannabis is authorized or recommended for use rather than prescribed, and patients are issued a card or designation which permits them to buy, use, possess, and, in some states, grow cannabis for personal medical use.^35^

## Evidence

THC-containing cannabis has been found in studies to be efficacious for treating nausea and vomiting, pain, insomnia, loss of appetite, and symptoms related to posttraumatic stress disorder.^15^ CBD alone showed some efficacy for social anxiety in a small trial as well as for seizure disorders, including a medication-sparing effect.^15^,^36^ Use of CBD for seizure disorders must also consider the purity of preparation as well as potential interaction with the patient's current seizure medications.^36^ In 2017, the National Academies of Sciences, Engineering, and Medicine (NASEM) published a comprehensive report evaluating the evidence to date for medical use of cannabis, as well as recommendations for future research. They found that there is substantial evidence to support use of cannabis for chronic pain in adults, conclusive evidence that oral cannabinoids are an effective antiemetic for chemotherapy-induced nausea and vomiting, and limited evidence that cannabis and oral cannabinoids are effective in increasing appetite and weight loss associated with HIV/AIDS.^37^ They also noted that there is substantial evidence that oral cannabinoids are an effective treatment for improving patient-reported multiple sclerosis spasticity symptoms and limited evidence that THC capsules improve symptoms of Tourette syndrome.^37^ They further identified many gaps in research and knowledge including efficacy for symptoms such as insomnia, which is measured as a primary symptom rather than a secondary outcome.^38^ Using cannabis to achieve abstinence or reduction in use of opioids or other substances is an area of great interest to researchers and patients which was not supported in the 2017 report.^38^ (See *Levels of evidence of efficacy of cannabis for select conditions*.)

Since the publication of the NASEM report in 2017, several additional systematic reviews and meta-analyses have examined the evidence for cannabis use with certain medical conditions. One concluded that noninhaled cannabis had a small to very small improvement in pain relief, physical functioning, and sleep quality among patients with chronic pain, along with several transient adverse reactions, compared with placebo.^40^ A rigorous review found that the potential benefits of cannabis-based medicine (herbal cannabis, plant-derived or synthetic THC, THC/CBD oromucosal spray) in chronic neuropathic pain might be outweighed by the potential harms and was additionally critical of the exclusion of patients with significant comorbidities and/or a history of substance use disorder from studies.^41^ A 2021 review reiterated that there is very low certainty evidence regarding the opioid-sparing effects of cannabis for chronic pain.^42^ Lack of even low- to moderate-quality evidence of efficacy of cannabis for multiple chronic conditions including symptoms of Parkinson disease has been noted.^43^ The authors of an extensive literature review mapped 11 approved medical conditions to available evidence published between 2016 and 2019.^44^ In a review of 198 studies, they again reiterated the lack of high-quality clinical trials but noted that trials evaluating the use of cannabis in multiple sclerosis, epilepsy, and chronic noncancer pain were of the highest quality and/or precision of measurement.^44^ They also noted that certain dosage forms and routes of administration had a favorable impact on risk-benefit ratios for epilepsy and chronic noncancer pain.^44^

**Table:** Levels of evidence of efficacy of cannabis for select conditions^3^,^9^

Levels of evidence of efficacy	Conclusive or substantial evidence	Moderate evidence	Limited evidence	Insufficient evidence
Benefits	Adult chronic pain Multiple sclerosis (MS)/spasticity Chemotherapy-induced nausea/vomiting Intractable seizures Dravet and Lennox-Gastaut syndromes (CBD)	Sleep disturbances related to pain, MS, fibromyalgia, sleep apnea Decreasing intraocular pressure in glaucoma	Dementia Parkinson disease Schizophrenia symptoms PTSD symptoms Appetite/weight issues with HIV/AIDS Traumatic brain injury Anxiety (CBD) Tourette syndrome	Depression Addiction abstinence IBS symptoms Cancer treatment Cancer-associated anorexia ALS symptoms Dystonia

Source: Clark CS, American Cannabis Nurses Association. *Cannabis: A Handbook for Nurses*. Wolters Kluwer; 2021.

Abbreviations: ALS, amyotrophic lateral sclerosis; CBD, cannabidiol; IBS, irritable bowel syndrome; PTSD, posttraumatic stress disorder.

The current federal status of cannabis as a prohibited, Schedule I drug creates significant barriers to clinical research. The impact of regulation of cannabis as opposed to strict prohibition is just beginning to be understood from a public health standpoint. The lack of controlled trial evidence specific to medical cannabis efficacy should therefore not be interpreted as indicating a lack of potential efficacy. Prospective observational studies have been done to evaluate the efficacy of cannabis for medical conditions. One such study used quantified scales and prescription drug monitoring data to correlate pain reduction, controlled substance use reduction, and well-being among patients with chronic orthopedic pain and medical cannabis authorization in the Pennsylvania medical cannabis program.^45^ Over the course of 12 months, patients experienced significant reduction in pain and improvement of function and quality of life, and 73% either ceased or reduced their consumption of opioids.^45^ Among patients with chronic back pain, medical cannabis has been correlated with improved pain and disability scores as well as with decreased use of opioids for both those taking less than 15 morphine milligram equivalents (MME) daily and more than 15 MME daily.^46^ The challenge of evaluating studies on medical cannabis includes how adverse reactions were measured and whether they were a primary outcome. As an example, pharmaceutical THC does not appear to impact appetite or sleep, which may either be a desirable quality or equated with ineffectiveness depending on the patient's condition and reason for use, while there is moderate evidence that pharmaceutical CBD can decrease appetite, which has been a known adverse reaction among children using it for seizure control but of little impact with adult users.^47^ Nonclinical trial evidence (also known as “real-world evidence”), such as that generated from medical and insurance records, is now being integrated into scientific studies of cannabis as well as its regulatory frameworks.^48^ Patient-generated evidence and perspectives can also help drive further study as well as be used in patient decision-making processes.^49^

## Dosing

The status of cannabis as a Schedule I drug on a federal level in the US also prohibits the prescribing of cannabis for routine medical use. Because cannabis is not a prescription, the typical instructions which would guide patients in use, such as dosage or labeling, are not required in cannabis authorizations in the US. In countries where cannabis is nationally legal, providers are required to include certain information on authorizations, such as maximum daily quantity (see: www.canada.ca/content/dam/hc-sc/migration/hc-sc/dhp-mps/alt_formats/pdf/marihuana/info/Medical-Document-EN.pdf for an example). Canadian dosing guidelines stress the individual nature of all cannabis dosing. As general guidance, they suggest that patients who are cannabis-naive start with no more than 1 mg of THC per dose.^50^ Adverse reactions to cannabis are primarily attributed to its THC content, the dose-equivalent of which should generally be limited to a self-titrated amount of a maximum of 30 mg daily, in conjunction with CBD.^19^ The variety of administration methods for cannabis can make the determination of accurate THC and CBD content challenging. MacCallum and Russo provide guidance based upon route of administration factors for smoking/vaporization, oral, oromucosal, and topical administration, though the latter is variable and primarily used for localized conditions only.^19^ A prospective long-term study in Canada assessing the safety of cannabis use for chronic noncancer pain in which an herbal cannabis product (12.5% THC) was dispensed to one group (n = 215) found that, on average, patients were dosing the dried herbal cannabis with minimal adverse reactions at 2.5 g/day.^51^ Oral preparations are easier to titrate for patient instruction and consistency.^19^ The authors suggest an initial regimen as below for oral THC products:

- Days 1-2: 2.5 mg THC-equivalent at bedtime. (May start at 1.25 mg for young patients and older adults or if other concerns are present.)
- Days 3-4: if the previous dose was tolerated, increase by 1.25-2.5 mg THC at bedtime.
- Days 5-6: continue to increase by 1.25-2.5 mg THC at bedtime every 2 days until the desired effect is obtained. In event of adverse reactions, reduce to the previous, best-tolerated dose.^19^

CBD dosing for seizure disorders is much higher than the dosage used in conjunction with THC or alone for other conditions. Pharmaceutical CBD (Epidiolex) is dosed at 5 mg/kg twice daily or more. By comparison, CBD used for other medical conditions or in conjunction with THC may be effective at 5-20 mg daily and may also balance THC effects.^19^

## Benefits

As noted, the clinical benefits of cannabis are evident for many patient symptoms, particularly chronic pain, but evidence standards at this writing include observational, preclinical, case studies, patient report and survey, and other less rigorous evidence due in part to both the legal status and stigma associated in the US with cannabis. Even in countries, such as Israel, where medical cannabis is legal and clinically available for medical use, patients report a feeling of stigma which may have subsequently delayed their use of cannabis for their symptoms.^52^ Reported benefits of cannabis include use for pain, nausea, muscle spasm, inflammatory conditions, seizure disorders, and gastrointestinal conditions.^53^ The potential for adjunctive treatment with established medication regimens, or for a reduction in adverse reactions or burden from current medications, is of clinical consideration in otherwise difficult-to-treat chronic conditions such as inflammatory bowel syndrome and fibromyalgia.^19^,^54^ Topical use of cannabis, primarily for its analgesic and anti-inflammatory effects, was reported by patients in a survey in Canada for a multitude of conditions, including atopic dermatitis, joint stiffness and pain, acne, and headaches.^55^ While promising and theoretically valid, the use of cannabinoids topically for dermatologic conditions is not yet clinically validated.^56^

## Risks

Common clinical adverse reactions to THC-containing cannabis products include mood disturbances such as anxiety and panic attacks; cognitive and central nervous system alterations; increased heart rate and cardiovascular symptoms; and respiratory effects from inhaled products.^27^ Adverse reactions from THC are dose-related and self-limiting, and respiratory depression cannot occur with cannabis as with opioids.^19^ However, there are clearly patients for whom cannabis is contraindicated, particularly THC-containing preparations. Cannabis cannot be advised at this time for pregnant or lactating patients; for patients with a history of or risk for psychosis; patients with cardiac conditions; and patients with cancer who are receiving immunotherapy treatments. Adolescents should likely avoid regular high-dose THC unless their medical needs currently outweigh long-term risks.^19^ Adverse reactions to CBD in pharmaceutical formulation include somnolence, sedation, lethargy, and fatigue, as well as elevation of liver transaminases; however, these effects are also dose-dependent and cannot be extrapolated to lower doses used for other conditions.^34^ One of the primary risks of CBD is that it is currently available in multiple over-the-counter products with little regulation or standardization of product.^34^ Additional risks from cannabis include potential harm from contaminants, such as microbes, heavy metals, and pesticides, which may be inhaled or ingested.^57^

## Harm reduction

There are two areas of harm reduction and cannabis which have interested public health researchers and professionals. The first is how to reduce potential harm from cannabis use, whether medical or recreational.^58^ These strategies include avoidance of use in youth; using low-potency THC or balanced THC/CBD chemovars; abstaining from synthetic nonpharmaceutical products; avoiding combustion and deep inhalation administration methods; avoiding high-frequency use and driving while impaired by cannabis; and avoidance of use by groups at high risk for misuse or with medical contraindication.^58^ Use of legal/regulated cannabis products where possible was added as a recommended strategy for consumption harm reduction in 2022 by public health experts, influenced by the increasing availability of such products.^59^ A clinical history of patient use of cannabis should therefore include questions regarding how and where cannabis was obtained in addition to general risk assessment and counseling.

The second is whether and how cannabis may be used to reduce harm from other medications or substances including prescription drugs, alcohol, tobacco, and opioids.^60^-^62^ When substituted for alcohol, there is also a difference in use for medical versus nonmedical cannabis users, with medical users reporting fewer drinks consumed on most days when cannabis was used while nonmedical users increased their consumption of alcohol on cannabis use days.^63^ There is compelling evidence, primarily from self-report survey and observational studies, that medical cannabis can lower consumption of opioids, antidepressants, alcohol, and tobacco.^62^ However, there are many limitations to measuring the clinical impact of this substitution including its measurement and its persistence as well as the impact of statewide policy and legalization.^64^

## Stigma and bias

Whether or not NPs authorize cannabis, they need to be aware of its potential impact on their patients and encourage open communication. Shared decision-making techniques can be used for cannabis as with any medication or medical decision.^49^ However, the stigma of cannabis use, even with medical authorization and indication, can limit both the practitioner and the patient from such information sharing.^65^ There is also evidence that cannabis stigma is racialized, both in the media and in policy application to patients.^65^ In light of patients being denied transplants and other services because of medically authorized cannabis, states such as New Mexico have codified that a qualified patient's use of cannabis “shall be considered the equivalent of the use of any other medication under the direction of a physician and shall not be considered to constitute the use of an illicit substance or otherwise disqualify a qualified patient from medical care.”^66^

The lack of insurance coverage for cannabis for medical conditions is also a significant barrier to its use and legitimate acquisition by patients, with 70% of patients in a national survey stating that cost is very prohibitive or entirely prohibitive, and many patients paying up to $350 or more for yearly examination and registration before being able to purchase cannabis from a medical dispensary.^67^ These factors make access to a legal and regulated market challenging for many patients, which in turn means patients may be less able or likely to use appropriately labeled and content-tested cannabis and may instead purchase it from the unregulated market.

## Nursing practice and policy

Ability to authorize cannabis is typically implemented in legislative statute and interpreted in policy or regulation.^1^ National nursing organizations have clear statements that either support patient choice in using cannabis or specify the obligation of the nurse to be aware and knowledgeable of medical cannabis in order to provide accurate patient counsel and care.^10^,^68^ Each state licensing board with jurisdiction over NP practice will use existing policies and rules to interpret how and whether the NP may recommend or manage cannabis medically. NPs are advised to verify with their individual licensing boards the parameters of their practice related to medical cannabis. Employers have no obligation to accommodate medical cannabis and its use for patients, and those seeking authorization should be informed that authorization does not provide this assurance in most states.^69^ In order to be prepared for state and national changes regarding cannabis and its use medically, professional associations which focus on the clinician role have been active for the last decade or more (Society of Cannabis Clinicians; American Cannabis Nurses Association) and may be a resource for ongoing education. In summary, NPs are likely to encounter patients who use or ask about cannabis, and preparation to discuss what is known and not known is a logical part of any health assessment. Ongoing self-education and assessment of both clinical and patient-provided evidence is a key part of any shared decision-making discussion, including those regarding cannabis.^49^
